# Postoperative hyperammonemic encephalopathy due to unexpected constipation in a patient with hyperornithinemia-hyperammonemia-homocitrullinuria syndrome: a case report

**DOI:** 10.1186/s40981-024-00726-z

**Published:** 2024-06-21

**Authors:** Haruka Tachibana, Nobuhiko Ohashi, Gaku Okumura, Ryusuke Tanaka, Satoshi Fuseya, Sayako Gotoh, Takashi Ishida, Sari Shimizu, Mikito Kawamata, Satoshi Tanaka

**Affiliations:** 1 Department of Anesthesiology and Resuscitology, Shinshu University School of Medicine, 3-1-1, Asahi, Matsumoto, Nagano, 390-8621 Japan; 2 Department of Medicine (Neurology and Rheumatology), Shinshu University School of Medicine, 3-1-1, Asahi, Matsumoto, Nagano, 390-8621 Japan

**Keywords:** Hyperornithinemia-hyperammonemia-homocitrullinuria syndrome, Urea cycle disorder, Hyperammonemia, Postoperative constipation

## Abstract

**Background:**

Hyperornithinemia-hyperammonemia-homocitrullinuria (HHH) syndrome is a rare autosomal recessive urea cycle disorder associated with a high risk of exacerbation of hyperammonemia during the perioperative period. Here, we describe an adult patient with HHH syndrome who developed hyperammonemic encephalopathy secondary to postoperative constipation.

**Case presentation:**

A 52-year-old patient with HHH syndrome underwent intrathecal baclofen pump insertion for lower limb spasticity under general anesthesia. The surgery was uneventful, without any increase in serum ammonia levels. However, after surgery, he was constipated, and on postoperative day (POD) 3, he fell into a coma with an exacerbation of hyperammonemia (894 µg/dL). After administering a glycerin enema, he defecated, leading to a rapid decrease in serum ammonia levels to 165 µg/dL. He regained consciousness, and serum ammonia levels remained stable as long as he defecated.

**Conclusions:**

We suggest strict management of defecation during the perioperative period to prevent hyperammonemia in patients with HHH syndrome.

## Background

Hyperornithinemia-hyperammonemia-homocitrullinuria (HHH) syndrome, which commonly presents with shared characteristics such as spastic paresis, epilepsy, and hyperammonemia, is an extremely rare autosomal recessive urea cycle disorder (UCD) caused by a deficient mitochondrial ornithine transporter [1, 2]. There are only a few case reports on the anesthetic experience of patients with HHH syndrome [3, 4], and optimal perioperative management for these patients has not yet been established. However, it is essential to prevent perioperative hyperammonemia. Perioperative conditions such as constipation, infection, gastrointestinal bleeding, hypokalemia and dehydration may affect ammonia production and clearance [5]. Herein, we present a case of an adult patient with HHH syndrome who developed hyperammonemic encephalopathy, mainly due to 3-day-long postoperative constipation.

## Case presentation

A 52-year-old male (height, 160 cm; weight, 50 kg; body mass index, 19.5 kg/m^2^) with intellectual disability was scheduled to undergo intrathecal baclofen pump insertion under general anesthesia to treat lower limb spasticity. Five years previously, the patient was diagnosed with HHH syndrome by genetic testing after recurrent epileptic seizures and hepatic encephalopathy. The patient was treated with lactulose (36 g/day), magnesium oxide (990 mg/day), and lacosamide (100 mg/day) for constipation and epilepsy and regularly consumed a low-protein diet supplemented with arginine (6 g/day). His serum ammonia levels were normally around 40–60 µg/dL (normal value, 12–66 µg/dL), with occasional elevations (100–150 µg/dL). The patient had undergone a colectomy for sigmoid cancer under general anesthesia without any complications at another hospital one year previously. Preoperative laboratory data showed normal liver and kidney function, and the serum ammonia level was 44 µg/dL. A preoperative diet (36 kcal/kg/day, 1.2 g/kg/day of protein) was provided until an evening meal the day before surgery. Fluid intake was maintained for up to 3 h before surgery, and lactulose, lacosamide, and arginine were continued until the morning of surgery.

In the operating room, the patient was monitored with electrocardiography, invasive arterial blood pressure, pulse oximeter, capnography, bladder temperature, and bispectral index. General anesthesia was induced with propofol 50 mg and fentanyl 50 µg. Thirty milligrams of rocuronium were administered to facilitate tracheal intubation. Anesthesia was maintained with oxygen 45%, desflurane 4%, and remifentanil 0.10–0.16 µg/kg/min. Hemodynamics were maintained with an occasional bolus of ephedrine 4 mg and continuous infusion of phenylephrine 0.3–0.5 mg/h. Sodium bicarbonate Ringer solution was administered during anesthesia. The surgery was completed without any complications. Intrathecal baclofen was administered at a dose of 20 µg/day. Nine minutes after discontinuation of anesthesia, the patient regained consciousness, and the tracheal tube was removed in the operating room. The duration of surgery and anesthesia was 106 and 228 min, respectively. The total intake was 1200 mL of sodium bicarbonate Ringer solution. During anesthesia, serum ammonia levels ranged from 27 and 46 µg/dL, and blood glucose levels ranged from 100 to 133 mg/dL. The patient was transferred to a high-care unit for postoperative monitoring.

**Fig. 1:**
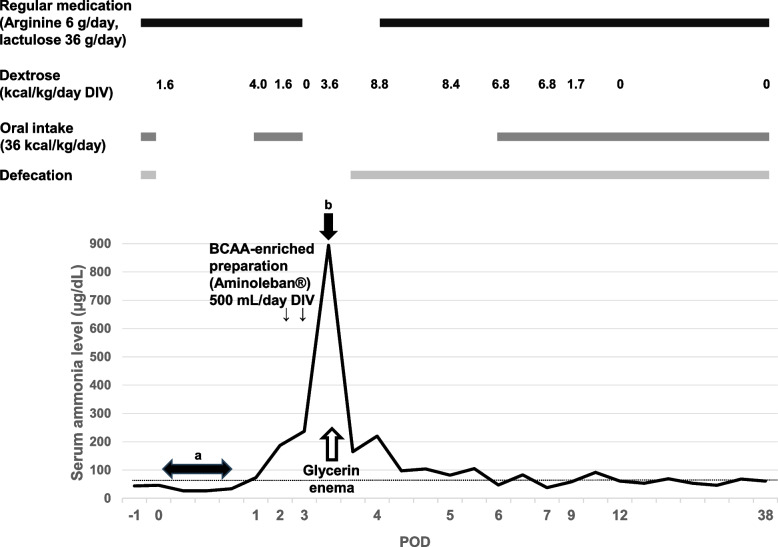
Serum ammonia levels and perioperative treatment. Serum ammonia levels ranged from 27 µg/dL to 46 µg/dL during anesthesia (**a**). Serum ammonia level increased to 894 µg/dL on POD 3 (**b**). Treatment included glycerin enema, intravenous glucose supplementation, cessation of protein intake, and control of defecation. Consequently, the serum ammonia level decreased to 165 µg/dL 1 h after defecation. The horizontal dotted line indicates a normal serum ammonia level (66 µg/dL). BCAA, branched-chain amino acids; POD, postoperative day

The serum ammonia levels during the postoperative period are shown in Fig. 1. On postoperative day (POD) 1, the serum ammonia level mildly increased to 73 µg/dL. There were no symptoms suggestive of metabolic decompensation, such as nausea, vomiting, poor appetite, or decline in consciousness. Postoperative pain was well-controlled with acetaminophen 300 mg/day and loxoprofen 60 mg/day. The patient was transferred to the general ward, and a low-protein diet and regular oral medications were recommenced on the morning of POD 1. In addition to his medication, sodium picosulfate solution was commenced because he experienced flatus but had no defecation. Maintenance solution (43 g/L dextrose) and dextrose-free Ringer solution were administered at 40 mL/h. On POD 2, the patient consumed 80% to 100% of his diet, although defecation was not observed. The patient felt nauseous, and a flapping tremor was observed as his serum ammonia level increased to 187 µg/dL in the afternoon. Therefore, an intravenous infusion of a branched-chain amino acid (BCAA)-enriched preparation (Aminoleban®) (500 mL/day) was initiated at 150 mL/h.

On the morning of POD 3, the patient was conscious; however, he was unable to eat due to worsening nausea. The diet was stopped, and dextrose-supplemented Ringer acetate solution (50 g/L dextrose) was administered. Intravenous infusion of a BCAA-enriched preparation (500 mL/day) was administered again in the afternoon. The patient vomited several times in the evening and became increasingly somnolent and agitated. The Glasgow Coma Scale (GCS) score was E3V2M5. Laboratory results showed an acute exacerbation of hyperammonemia (894 µg/dL). Arterial blood gas analysis on room air showed anion-gap metabolic acidosis and compensatory respiratory alkalosis. Computed tomography of the head showed no cerebral edema. The patient fell into a deep coma (GCS E2V3M2) and was admitted to the intensive care unit. The patient received intravenous hydration supplemented with dextrose and lipid emulsion. Furthermore, the patient received a glycerin enema and defecated. One hour after defecation, the serum ammonia level rapidly decreased to 165 µg/dL, and the state of consciousness improved (GCS E3V3M5). On POD 4, the patient remained somnolent. Therefore, the medication was resumed via a nasogastric tube. To control defecation, which was intentionally managed to induce diarrhea, magnesium oxide (1650 mg/day) and sodium picosulfate solutions were administered via a nasogastric tube. The serum ammonia level fluctuated between 97 and 220 µg/dL, depending on the defecation status. On POD 6, the serum ammonia level normalized, and his mental status improved (GCS E4V5M6). The patient was transferred to the general ward on POD 7, and defecation management and rehabilitation were continued. Subsequently, the patient’s clinical course was uneventful, and discharged on POD 45 without any complications.

## Discussion

A patient with HHH syndrome was first reported in 1969, in which intermittent hyperammonemia was associated with persistent hyperornithinemia and homocitrullinuria [6]. The estimated incidence of all UCDs is 1:35000 live births, and HHH syndrome accounts for 1% to 3.8% of all UCDs [1]. Clinical symptoms include acute encephalopathy secondary to hyperammonemia, epilepsy, spastic paresis, intellectual disability, and chronic liver dysfunction. There is a large variability in the age of onset, clinical manifestations, and outcomes among individuals. However, patients with HHH syndrome often live a normal lifespan after treatment and can have surgery and general anesthesia. As well as other UCDs, special concerns should be addressed to prevent perioperative hyperammonemia.

Hyperammonemia is exacerbated in patients with UCDs due to the ingestion of protein-rich foods and may lead to vomiting, lethargy, or even episodes of coma. As serum ammonia levels can increase in patients with UCDs in a state of hypercatabolism caused by fasting and surgical stress after surgery [7], a low-protein diet should be resumed as early as possible in patients with UCDs [8], similar to our patient. Postoperative constipation is another critical factor that leads to hyperammonemia. The gastrointestinal tract is the primary site of ammonia production and absorption into the blood stream, as shown by the fact that the concentrations of ammonia in the portal vein are approximately three times higher than those in the systemic blood [9]. In healthy individuals, most intestinal ammonia is removed by the urea cycle and does not contribute to elevated serum ammonia levels. However, severe constipation for over a week has been reported to lead to hyperammonemic encephalopathy, even in patients without UCDs [10]. In contrast, elevated intestinal ammonia levels directly lead to hyperammonemia in patients with UCDs, including those with HHH syndrome. Therefore, constipation for only three days could have resulted in hyperammonemia in this patient, particularly when the diet, including nitrogen, was resumed soon after surgery. We considered constipation as the main cause of hyperammonemia exacerbation in this patient because his serum ammonia level rapidly normalized after a glycerin enema and was maintained at his usual defecation level. Additionally, BCAA-enriched preparations, which are frequently used to treat hepatic encephalopathy, were ineffective in this patient and could have aggravated hyperammonemia as a nitrogen source. In cases of acute hyperammonemia, essential amino acids or BCAA are suggested to be reintroduced within 24 h [2] but should be used carefully in patients with HHH syndrome.

In conclusion, we present the case of an adult patient with HHH syndrome who developed postoperative hyperammonemia and encephalopathy mainly due to constipation. Postoperative patients with HHH syndrome should be monitored for their state of consciousness and serum ammonia levels under strict control of defecation, diet, and fluid replacement.

## Data Availability

Not applicable.

## Abbreviations

BCAA: Branched-chain amino acids
HHH syndrome: Hyperornithinemia-hyperammonemia-homocitrullinuria syndrome
POD: Postoperative day
UCD: Urea cycle disorder
GCS: Glasgow Coma Scale
