# Correction: Postoperative hyperammonemic encephalopathy due to unexpected constipation in a patient with hyperornithinemia-hyperammonemia-homocitrullinuria syndrome: a case report

**DOI:** 10.1186/s40981-024-00753-w

**Published:** 2024-11-14

**Authors:** Haruka Tachibana, Nobuhiko Ohashi, Gaku Okumura, Ryusuke Tanaka, Satoshi Fuseya, Sayako Gotoh, Takashi Ishida, Sari Shimizu, Mikito Kawamata, Satoshi Tanaka

**Affiliations:** 1 Department of Anesthesiology and Resuscitology, Shinshu University, School of Medicine, 3‑1‑1, Asahi, Matsumoto, Nagano, 390‑8621 Japan; 2 Department of Medicine (Neurology and Rheumatology), Shinshu University School of Medicine, 3‑1‑1, Asahi, Matsumoto, Nagano, 390‑8621 Japan

**Correction: JA Clin Rep 10, 42 (2024)**

**https://doi.org/10.1186/s40981-024-00726-z**

Following publication of the original article [1], it was reported that the following data in bold should be included in the article. Nobuhiko Ohashi and Gaku Okumura should be included in the author group as they provided treatments for hyperammonemia and disturbed consciousness.

**Previous Author group**

Haruka Tachibana1, Ryusuke Tanaka1*, Satoshi Fuseya1, Sayako Gotoh1, Takashi Ishida1, Sari Shimizu1, Mikito Kawamata1 and Satoshi Tanaka1

**Updated Author group**

Haruka Tachibana1, **Nobuhiko Ohashi2** and **Gaku Okumura2,** Ryusuke Tanaka1*, Satoshi Fuseya1, Sayako Gotoh1, Takashi Ishida1, Sari Shimizu1, Mikito Kawamata1 and Satoshi Tanaka1

**Affiliation**

**2 Department of Medicine (Neurology and Rheumatology), Shinshu University School of Medicine, 3‑1‑1, Asahi, Matsumoto, Nagano 390‑8621, Japan**

**Authors Contribution**

HT, RT, HF, and SS made clinical decisions. **NO and GO made clinical decisions and treated hyperammonemia.** HT and RT drafted the manuscript. SG and TI supervised the clinical decisions. MK and ST supervised the manuscript drafting. All authors have read and approved the final manuscript.

This has been corrected above and the original article has been updated.
